# Correlation of Triglyceride-Rich Lipoprotein Cholesterol and Diabetes Mellitus in Stroke Patients

**DOI:** 10.1155/2022/7506767

**Published:** 2022-11-04

**Authors:** Ruiqing Luo, Huifang Jiang, Tao Wang, Yanfang Xu, Xueliang Qi

**Affiliations:** ^1^Department of Neurology, The Second Affiliated Hospital of Nanchang University, Nanchang, Jiangxi, China; ^2^Department of Medical Record, Affiliated Stomatological Hospital of Nanchang University, Nanchang, Jiangxi, China; ^3^Center for Prevention and Treatment of Cardiovascular Diseases, The Second Affiliated Hospital of Nanchang University, Nanchang, Jiangxi, China

## Abstract

**Background:**

Previous studies have revealed that triglyceride-rich lipoprotein cholesterol (TRL-c) is closely related to diabetes mellitus (DM) in hypertensive patients. However, this relationship in stroke patients has not been reported. The aims of this study are to investigate the relationship between TRL-c and diabetes in adult Chinese stroke.

**Methods:**

Patients with stroke treated in the Department of Neurology of the Second Affiliated Hospital of Nanchang University from January 2019 to January 2021 were selected. TRL-c was calculated from total cholesterol minus (high-density and low-density lipoprotein). DM was diagnosed based on previous medical history (diagnosed by secondary hospitals or above) and/or current use of hypoglycemic drugs and/or intravenous blood glucose measurement (fasting blood glucose ≥7.0 mmol/L or nonfasting blood glucose > 11.1 mmol/L). The relationship between the TRL-c and DM was determined using multivariate logistic regression, smoothing curve fitting (penalized spline method), and subgroup analysis.

**Results:**

A total of 890 patients with stroke (age, 66.1 ± 11.8 years) were enrolled, including 329 females. Multivariate logistic regression analysis demonstrated that TRL-c had a positive association with DM (OR 1.88; 95% CI: 1.22 to 2.89). Strong linear associations of TRL-c with DM were confirmed by restricted cubic spline analysis. And the association between TRL-c and DM was consistent in the different subgroups.

**Conclusion:**

Positive associations were found between TRL-c and DM in patients with stroke.

## 1. Introduction

Diabetes mellitus (DM) is a major public health problem in the world. In 2020, the number of adults with DM in the Chinese mainland was estimated to be 129.8 million [1]. In addition, the proportion of undiagnosed DM is also high [2]. Consequently, it is of great significance to further explore the risk factors of diabetes for early diagnosis, intervention, and prevention of diabetes. Diabetes is closely related to dyslipidemia, one of its risk factors. Diabetes and dyslipidemia interact and serve as a common risk factor for atherosclerotic disease. A meta-analysis has shown that although statin therapy can reduce low-density lipoprotein cholesterol, the residual risk of atherosclerotic cardiovascular disease (ASCVD) in diabetic patients is high [3]. As a result, triglyceride-rich lipoprotein cholesterol (TRL-c) is an essential factor leading to residual cardiovascular disease risk and is a newly emerging index for cardiovascular disease risk assessment [4]. TRL-c mainly includes very low-density lipoprotein, intermediate-density lipoprotein, and nonfasting chylomicrons. In addition, studies have demonstrated that TRL-c is closely related to insulin resistance [5], obesity [6], and abnormal glucose tolerance [7].

Moreover, a positive correlation between TRL-c and diabetes has been investigated in recently published studies [8, 9]. However, the results obtained from another study suggested no correlation [10]. The correlations reported in relevant studies are different and the populations included are only hypertensive patients, females, and public officials. In addition, no research has been conducted on stroke patients with ASCVD events. Therefore, this study aims to report the association between TRL-c and diabetes in patients with stroke to provide relevant evidence for secondary diabetes prevention.

## 2. Methods

### 2.1. Participants

**Figure 1:**
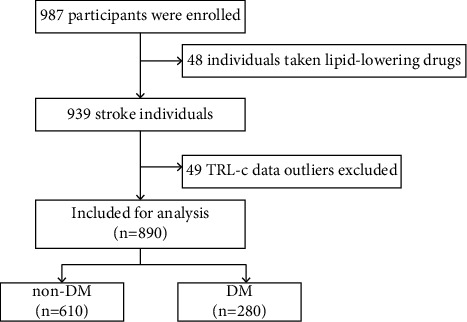
Flow chart of study participants.

Based on The China National Stroke Screening and Prevention Project (CNSSPP) [11]. 987 Patients with stroke were treated in the Department of Neurology of the Second Affiliated Hospital of Nanchang University from January 2019 to January 2021 and were retrospectively analyzed in this study. The inclusion criteria were as follows: (1) patients with stroke (including cerebral infarction, intracerebral hemorrhage, and subarachnoid hemorrhage) as the first diagnosis and first onset (the same type of diseases); (2) age ≥18 years; (3) signed informed consent. The exclusion criteria were as follows: (1) asymptomatic lacunar infarction, multiple lacunar infarctions; (2) previous stroke history (the different types of diseases were not excluded, such as patients with previous intracerebral hemorrhage suffering from cerebral infarction could be included); (3) traumatic intracerebral hemorrhage, subdural/epidural hemorrhage, traumatic subarachnoid hemorrhage, unruptured aneurysm, transient ischemic attack, and others. After excluding 48 patients who previously took blood pressure-lowering drugs and 49 patients with TRL-c outliers, 890 patients with acute stroke were included in this study (Figure 1). This study followed the principles of the Declaration of Helsinki and all participants received information on the study and provided written informed consent to participate.

### 2.2. Data Collection

The basic data were collected through face-to-face interviews, physical measurements, laboratory examinations, and auxiliary examinations. (1) Basic information: demographic information, contact information, and conditions in this study. The cause of death and relevant information were registered if the subject died. (2) Lifestyle: stroke risk factors, such as smoking, drinking, lack of exercise, and their changes were determined. (3) History: stroke history, hypertension, diabetes, atrial fibrillation, dyslipidemia, and treatment and control were identified. (4) Physical examination: height, body weight, and blood pressure. Professionals determined whether the subjects had regular rhythms by using cardiac auscultation. Atrial fibrillation was detected by electrocardiography (ECG) for patients with irregular rhythms. (6) Laboratory examination: fasting plasma glucose, blood lipid, glycosylated hemoglobin, and homocysteine were measured.

### 2.3. Outcome Definition

Hypertension was defined as systolic blood pressure ≥ 140 mmHg and/or diastolic blood pressure ≥ 90 mmHg, with a history of hypertension (three times on different days), and/or currently using antihypertensive medications [12]. Atrial fibrillation was defined as previous medical history (diagnosed by secondary hospitals or above) and/or ECG displaying the disappearance of the P wave, replaced by an irregular F wave and irregular RR intervals [13]. Smoking status was classified into “nonsmoker” and “current smoker” (a current smoker is one who smokes at least one cigarette a day for more than six consecutive or cumulative months). Alcohol use status was categorized into “nondrinker” and “current drinker” (current drinker is one who drinks wine ≥3 times/week, ≥ 100 g/time). TRL-c: TRL-c = total cholesterol (TC)-low-density lipoprotein (LDL)-high-density lipoprotein (HDL) [14].

### 2.4. Definition of DM and Stroke

DM was defined as previous medical history (diagnosed by secondary hospitals or above), current use of hypoglycemic drugs, or intravenous blood glucose measurement. (1) Diabetic symptoms: fasting blood glucose ≥7.0 mmol/L (126 mg/dl) or nonfasting blood glucose >11.1 mmol/L. (2) No typical diabetic symptoms: fasting blood glucose ≥7.0 mmol/L at least two times or nonfasting blood glucose >11.1 mmol/L [2]. A stroke was defined as a sudden clinical sign of persistent focal or total neurological deficit due to the blockage or rupture of the arterial system. In addition, this defect has symptoms or signs lasting for more than 24 h (unless death), or CT or Magnetic resonance imaging (MRI) confirmed deficit consistent with acute stroke after excluding other nonvascular causes (such as primary brain tumor, brain metastasis, postseizure paralysis, brain trauma, infection, or other nonischemic causes). It is divided into ischemic stroke and hemorrhagic stroke. Ischemic stroke refers to cerebral infarction, a neurological deficit caused by cerebral vascular stenosis or obstruction. Its symptoms can last at least 24 h, or a new infarct can be confirmed by imaging. It is caused by focal symptoms and signs of the nervous system are consistent with the affected vessels' blood supply. Hemorrhagic stroke, including intracerebral and subarachnoid hemorrhage, damages brain tissue caused by the rupture of the cerebral vessels. Intracerebral hemorrhage is defined as primary nontraumatic intraparenchymal hemorrhage. Subarachnoid hemorrhage refers to the blood flowing into the subarachnoid space after the rupture of blood vessels at the bottom or on the surface of the brain, causing corresponding clinical symptoms [15].

### 2.5. Statistical Analysis

The statistical analysis was performed using Empower 2.0 (R) (https://www.empowerstats.com) and the statistical package R (https://www.rproject.org, version 4.1.2). The measurement data following the normal distribution were expressed as mean ± standard deviation (*X* ± *S*) and compared by the *t*-test between groups. The measurement data inconsistent with the normal distribution were expressed as the median (*P*_25_–*P*_75_) and compared with the Mann–Whitney test between groups. The enumeration data were expressed as *N*(%) and compared using the *χ*^2^ test between groups. The nonconditional logistic regression models (represent results by odds ratio(OR) and 95% CI) for major covariables adjusted for the main covariates in three models were designed to assess the independent association of the TRL-c level with DM. The covariates were selected on the basis of statistical significance in the univariable analyses, their clinical importance, and the estimated variables change of at least 10% of potential confounding effects. The penalized spline regression method (a fitted smoothing curve) and the generalized additive model (GAM) conducted the dose-response relationship for the TRL-c level with DM. In addition, we conducted subgroup analyses to explore the potential factors modifying the association. *P* < 0.05 was considered statistically significant.

## 3. Results

### 3.1. Baseline Characteristics

A total of 890 patients with acute stroke (mean age: 66.7 ± 11.8 years; 37.0% females) were enrolled. There were 280 diabetic patients, with an incidence of 31.4%. Compared with the non-DM group, patients with DM had high values of SBP, NIHSS score, Fasting plasma glucose, triglyceride, TRL-c, and lower values for current smoking, HDL-c, serum homocysteine, the prevalence of hypertension, atrial fibrillation, antiplatelet drugs, and antihypertensive drugs, as shown in Table 1.

### 3.2. Univariate Analysis of DM and Common Risk Factors

Table 2 shows the univariate analysis results of age, gender, smoking, drinking, BMI, SBP, DBP, TRL-c, homocysteine, hypertension, atrial fibrillation, and antihypertensive drugs. We found that smoking, BMI, SBP, TRL-c, homocysteine, history of hypertension, atrial fibrillation, and antihypertensive drugs were significantly associated with DM (*P* < 0.05).

### 3.3. Association of TRL-c with Elevated DM

**Figure 2:**
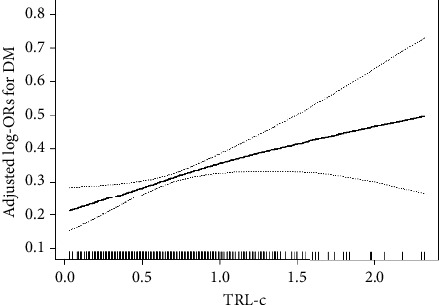
Dose-response association between TRL-c and DM. All were adjusted for age, gender, BMI, current smoking, SBP, Hcy, history of hypertension and atrial fibrillation, and antihypertensive drugs.

After adjusting for variables including age, gender, BMI, current smoking, SBP, homocysteine, history of hypertension, atrial fibrillation, and antihypertensive drugs, TRL-c was positively correlated with the risk of DM. DM risk increased by 88% per 1 mmol/L increase in TRL-c (OR = 1.88, 95% CI: 1.22–2.89, *P*  <  0.005).  Table 3 presents the correlation between TRL-c and diabetes using the multivariate logistic regression model. Models 1–3 are the original, slightly adjusted, and fully adjusted models. In the three models, the correlation between TRL-c and diabetes was consistent. After quartering TRL-c in the fully adjusted model (Model 3), the adjusted OR (95% CI) of diabetes in groups 2, 3, and 4 compared with group 1 were 1.22 (0.79, 1.87), 1.26 (0.82, 1.95), and 1.69 (1.11, 2.56), respectively (trend test, *P*  <  0.05), indicating a dose-response correlation between TRL-c and diabetes risk (Figure 2).

### 3.4. Subgroup Analysis

**Figure 3:**
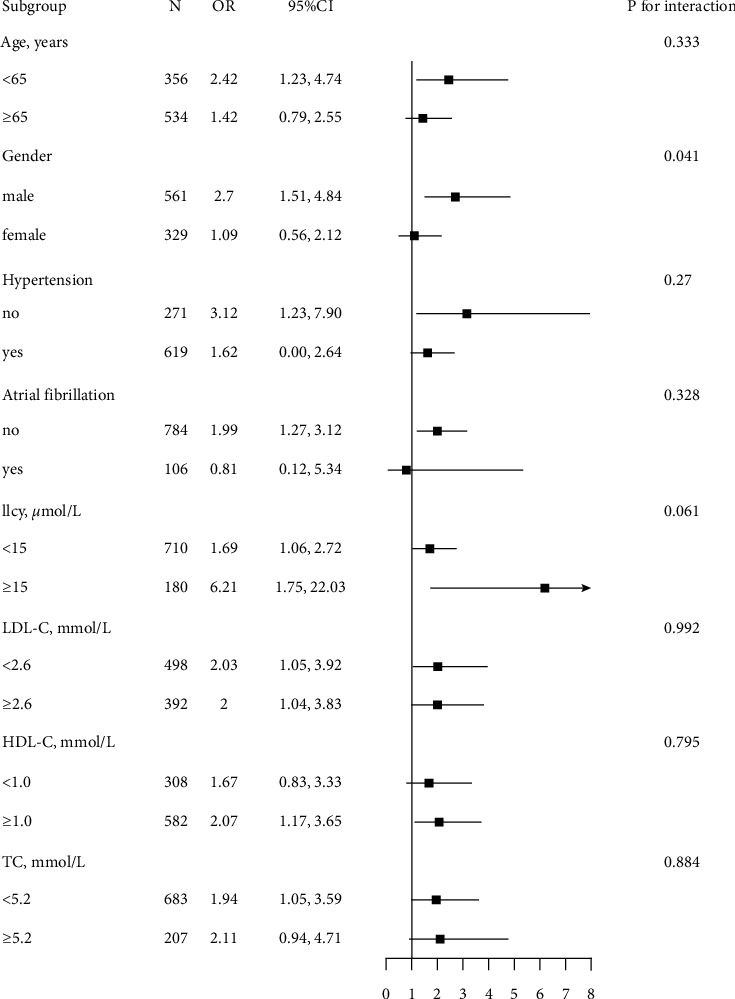
Subgroup analyses of the association between TRL-c and DM. Adjusted for age, gender, BMI, current smoking, SBP, Hcy, history of hypertension and atrial fibrillation, and antihypertensive drugs, if not stratified.

Stratified analyses were conducted to reveal the relationship between TRL-c (per 1 unit increment) and DM in different subgroups (Figure 3). No significant interactions were found in the following subgroups, including age (<64 vs ≥ 65 years), hypertension, atrial fibrillation, Hcy (<15 vs ≥ 15 *μ*mol/L), LDL-c (<2.6 vs ≥ 2.6 mmol/L), HDL-c (<1.0 vs ≥ 1.0 mmol/L), TC (<5.2 vs ≥ 5.2 mmol/L) subgroups (*P* values for >0.05 for all), whereas TRL-c had a significant relationship between male patients and DM (males: OR = 2.70, 95% CI 1.51–4.84; females: OR = 1.09, 95% CI 0.56–2.12; *P* for interaction = 0.041).

## 4. Discussion

The present study found that among the patients in our hospital with a stroke, the incidence of diabetes was 31.5% and TRL-c was positively correlated with DM risk, indicating that TRL-c may be a marker for diabetes risk. This new finding expands the application population compared with previous studies. Even after the occurrence of ASCVD events, TRL-c may provide a new idea for the prevention and treatment of diabetes.

The consensus statement of the European Atherosclerosis Society on triglyceride-rich lipoproteins and their residues in 2021 [16] mentioned that reducing triglyceride-rich lipoproteins and their residues is a strategic method for preventing ASCVD. There is increasing evidence that a high concentration of TRL-c residues is a cardiovascular disease risk. Over the past 30 years, epidemiological and genetic studies have confirmed that elevated plasma TRL-c and their residues are correlated with ASCVD events [17], including myocardial infarction [18], stroke [19], coronary artery calcification [20], aortic stenosis [21] and all-cause death [22, 23]. Moreover, some scholars [24] proposed that although it remains uncertain whether it can be used as a monitoring target for drug therapy, it should be detected in cardiovascular health screening and disease diagnosis.

In addition, increasing evidence links TRL-c to DM and other metabolic diseases. In the Woman's Genome Health Study (WGHS) [8], 15,813 participants in the baseline fasting state were analyzed, revealing that at the average 18.6-year follow-up, 1453 new diabetes cases occurred. In the Cox model with adjusted basic covariates, TRL-c significantly correlated with diabetes (HR = 1.39, 95% CI 1.28–1.51, *P*  <  0.001). Zhou Wei et al. [9] in China conducted a cross-sectional analysis of the data of 13,721 hypertensive patients without lipid-lowering drugs. They found that TRL-c significantly and positively correlated with diabetes risk (OR = 1.73, 95% CI: 1.54–1.94). This finding suggested that TRL-c may be a reliable diabetes marker and may provide a new strategy for the prevention and treatment of diabetes, consistent with our results. However, foreign scholars Carvalho et al. [10] included a Brazil Adult Health (ELSA-Brasil) cohort of 4463 participants. They showed that 366 new diabetes cases were found after an average of 3.7 years of follow-up. Also, their plasma TRL-c concentrations were not related to new diabetes risk and could not improve the prediction of diabetes. Studies have reported inconsistent conclusions on their correlation, so more studies are needed to evaluate the potential of TRL-c in preventing diabetes.

The mechanisms underlying the relationship between TRL-c and DM are not yet fully understood. DM is related to increased cardiovascular disease risk [25]. The increased incidence of atherosclerosis in people with diabetes has multiple causes. An important cause is the high incidence of specific dyslipidemia, the so-called “diabetic dyslipidemia” characterized by elevated TG, increased TRL-c, small and dense LDL particles, and reduced HDL. This pattern of dyslipidemia is prevalent in those with type 2 diabetes [17, 26]. In addition to the correlation between fasting serum triacylglycerol and diabetes, there is evidence that the plasma concentration of residual TRL-c and the cholesterol content in TRL reflect the pathophysiological process leading to diabetes progression [27, 28]. Therefore, residual lipoproteinemia should be a risk factor for targeted type 2 diabetes treatment [29, 30].

The strengths of our study include using standardized measurements for physical and laboratory tests and relatively large stroke population research. The present study still has limitations. First, although we carefully adjusted for potential confounders, we cannot entirely rule out the possibility of residual confounding by unmeasured factors, such as dietary variables, sleep duration, and physical activity. It is well known that the daily diet has a significant impact on diabetes and cerebrovascular diseases [31]. The study population was in the “oriental diet” pattern, and they often consumed beans, which contain isoflavones, which can have beneficial effects on diabetes and cardiovascular diseases. The relationship between soy products, isoflavones, diet, and CVD has become a controversial topic [32]. This question is undoubtedly interesting and opens up intriguing fields in the impact of diet on diabetes and cerebrovascular diseases. For example, studies have reported that one year of treatment with broom isoflavin improves surrogate endpoints associated with the risk of diabetes and cardiovascular disease in postmenopausal women with MetS [33]. It was worth mentioning that, A growing number of studies concerning gut microbiota and stroke. Recent studies have revealed that changes in gut microbiota ecology are related to cerebrovascular diseases [34, 35]. Gut dysbiosis may result from obesity, metabolic disorders, cardiovascular disease, and sleep disorders; Lack of physical activity is associated with gut dysbiosis as well. These may coexist in various patterns in stroke patients, enhancing the risk, incidence, and progression of cerebrovascular lesions, creating a vicious circle [36]. Meanwhile, studies have shown that the prognosis of stroke patients is significantly improved after the use of drugs that improve gut microbiota [37]. Therefore, the next research plan is to collect the factors affecting intestinal microbial homeostasis, dietary habits, sleep duration, physical activity, et al. Moreover, drugs or nutrients that can regulate and improve gut microbial community were collected, such as Probiotics/Prebiotics and Vit B12. Second, a cohort study based on data obtained from the medical records of patients visiting for routine clinical practice has been intentionally and systematically not been collected for research and therefore has several inherent limitations. Third, no distinction is made between types 1 and 2 diabetes in the database. Finally, the information on TRL-c subclasses was not complete in the dataset, so only TRL-c levels were selected as the exposure.

## 5. Conclusion and Future Directions

The present study found an independent positive association between TRL-c and DM in stroke patients. Our findings further support the possible new strategy of TRL-c for the prevention and treatment of DM.

It is worth mentioning that, medical treatment has been shifted to being more prophylactic as a recent trend. Meanwhile, it has been suggested that the alarming increase in the incidence of noncommunicable diseases, including obesity, cancers, diabetes, and cerebrovascular illnesses is the epidemiologic result of a nutrition transition characterized by dietary patterns [38]. It is well known that a healthy diet and physical activity can reduce the risk of noncommunicable diseases, including diabetes [39]. On the one hand, although Mediterranean-style eating patterns approaches have proven successful as part of the treatment for obesity and cardiometabolic derangements within clinical trial scenarios, they lack effectiveness in the long term, mainly due to poor compliance [40]. On the other hand, some studies have shown that Supplementation with probiotics or prebiotics may improve poststroke outcomes, as these supplements improved lipid and glucose metabolism in overweight people and those with diabetes mellitus [41]. Indeed, these results could provide a possible further strategy to prevent complications and delay the progression of Diabetes Mellitus. Some research [42] have thus turned its attention to nutraceutics, nutrients that have the ability to modulate physiological and pathophysiological molecular mechanisms, thus resulting in favorable health outcomes. In a word, preventing or retarding the onset of diseases has become a more attractive and cost-effective strategy in the medical arena of the prophylactic effects of dietary supplements and nutrients. The revised content is marked in red in the manuscript.

## Figures and Tables

**Table 1:** Clinical characteristics of the study population.

Variables	All patients (*n* = 890)	Non-DM (*n* = 610)	DM (*n* = 280)	*P* value
Age (years)	66.7 ± 11.8	66.9 ± 12.0	66.4 ± 11.4	0.538
Female (*n*) (%)	329 (37.0%)	215 (35.2%)	114 (40.7%)	0.117
Current smoking (*n*) (%)	128 (14.4%)	100 (16.4%)	28 (10.0%)	0.012
Current drinking (*n*) (%)	75 (8.4%)	57 (9.3%)	18 (6.4%)	0.146
BMI (kg/m^2^)	20.7 ± 1.5	20.6 ± 1.5	20.8 ± 1.5	0.073
SBP (mmHg)	143.4 ± 21.4	142.3 ± 21.2	145.7 ± 21.8	0.032
DBP (mmHg)	83.9 ± 13.0	83.5 ± 13.0	84.7 ± 13.0	0.206
NIHSS score	1.0(0–3.0)	1.0(0–3.0)	1.0(0–2.0)	0.002
Modified Rankin Scale score	1.4 ± 0.8	1.4 ± 0.8	1.4 ± 0.8	0.929
Fasting plasma glucose (mmol/L)	6.4 ± 2.7	5.4 ± 1.1	8.5 ± 3.7	<0.001
Total cholesterol (mmol/L)	4.4 ± 1.1	4.4 ± 1.1	4.5 ± 1.3	0.418
Triglyceride (mmol/L)	1.4 ± 1.0	1.3 ± 0.8	1.7 ± 1.2	<0.001
LDL-c (mmol/L)	2.5 ± 0.9	2.5 ± 0.9	2.6 ± 1.1	0.073
HDL-c (mmol/L)	1.1 ± 0.3	1.2 ± 0.3	1.0 ± 0.3	<0.001
TRL-c (mmol/L)	0.7 ± 0.3	0.7 ± 0.3	0.8 ± 0.3	<0.001
Serum homocysteine (*μ*mol/L)	13.2 ± 7.0	13.6 ± 7.2	12.3 ± 6.4	0.013
Hypertension (*n*) (%)	619 (69.6%)	390 (63.9%)	229 (81.8%)	<0.001
Dyslipidemia (*n*) (%)	425 (47.8%)	261 (42.8%)	164 (58.6%)	<0.001
Atrial fibrillation (*n*) (%)	106 (11.9%)	85 (13.9%)	21 (7.5%)	0.006
Antiplatelet drugs (*n*) (%)	790 (88.8%)	526 (86.2%)	264 (94.3%)	<0.001
Antihypertensive drugs (*n*) (%)	550 (61.8%)	356 (58.4%)	194 (69.3%)	0.002
Anticoagulant drugs (*n*) (%)	196 (22.0%)	345 (23.8%)	51 (18.2%)	0.063

Data are the mean ± SD, or number (percentage). BMI: body mass index; SBP: systolic blood pressure; DBP: diastolic blood pressure; NIHSS; modified Rankin Scale; HDL-c: high-density lipoprotein cholesterol; LDL-c: low-density lipoprotein cholesterol, TRL-c: triglyceride-rich lipoprotein cholesterol.

**Table 2:** Crude association of DM with common risk factors analyzed by univariate analysis.

	Statistics	OR (95%CI)	*P* value
Age (years)	66.7 ± 11.8	1.0 (0.98, 1.01)	0.538
Gender			
Male	561 (63.0%)	Ref	0.117
Female	329 (37.0%)	1.26 (0.94, 1.69)	
Current smoking			
No	762 (85.6%)	Ref	0.012
Yes	128 (14.4%)	0.57 (0.36, 0.88)	
Current drinking			
No	815 (91.6%)	Ref	0.148
Yes	75 (8.4%)	0.67 (0.38, 1.16)	
BMI (kg/m^2^)	20.7 ± 1.5	1.10 (1.00, 1.21)	0.043
SBP (mmHg)	143.4 ± 21.4	1.01 (1.00, 1.02)	0.032
DBP (mmHg)	83.9 ± 13.0	1.01 (1.0, 1.02)	0.206
TRL-c (mmol/L)	0.7 ± 0.3	1.99 (1.32, 3.01)	0.001
Serum homocysteine (*μ*mol/L)	13.2 ± 7.0	0.97 (0.94, 0.99)	0.016
Hypertension			
No	271 (30.4%)	Ref	<0.001
Yes	619 (69.6%)	2.53 (1.79, 3.58)	
Dyslipidemia			
No	465 (52.2%)	Ref	<0.001
Yes	425 (47.8%)	1.89 (1.42, 2.52)	
Atrial fibrillation			
No	784 (88.1%)	Ref	0.007
Yes	106 (11.9%)	0.50 (0.30, 0.83)	
Antihypertensive drugs			
No	340 (38.2%)	Ref	0.002
Yes	550 (61.8%)	1.61 (1.19, 2.17)	
Anticoagulant drugs			
No	694 (77.9%)	Ref	0.064
Yes	550 (22.0%)	0.71 (0.50, 1.02)	

BMI: body mass index; SBP: systolic blood pressure; DBP: diastolic blood pressure; TRL-c: triglyceride-rich lipoprotein cholesterol.

**Table 3:** Association between TRL-c and DM in different models (*n* = 890).

TRL-c (mmol/L)	Elevated DM OR (95% CI), *P* value								
	Model 1			Model 2			Model 3		
Per 1 mmol/L increase	1.99	1.32∼3.01	<0.001	1.93	1.28∼2.93	0.002	1.68	1.08∼2.60	0.021
Quartiles									
Q1 (<0.50)	Ref			Ref			Ref		
Q2 (0.51–0.70)	1.12	0.74∼1.69	0.598	1.11	0.73∼1.68	0.629	1.22	0.79∼1.88	0.361
Q3 (0.71–0.91)	1.18	0.78∼1.78	0.440	1.15	0.76∼1.74	0.513	1.28	0.83∼1.98	0.267
Q4 (>0.92)	1.71	1.15∼2.54	0.008	1.67	1.12∼2.49	0.012	1.54	1.01∼2.35	0.045
*P* for trend	0.008			0.012			0.012		

Model 1: adjusted for none. Model 2: adjusted for age and gender. Model 3: adjusted for age, gender, BMI, current smoking, SBP, Hcy, history of hypertension, atrial fibrillation, dyslipidemia, and antihypertensive drugs.

## Data Availability

The datasets used and/or analyzed during the current study are available from the corresponding author upon reasonable request.
